# Improving market infrastructure for small entrepreneurship in Uzbekistan: a sociological opinion on export potential, employment, and social inclusion

**DOI:** 10.3389/fsoc.2026.1926726

**Published:** 2026-10-01

**Authors:** Firuza Saidkulova, Dilafiruz Narzieva, Nigina Chorieva, Firuza Achilova, Jasurbek Jonibekov, Mokhichekhra Rajabova

**Affiliations:** 1 Bukhara State University, Bukhara, Uzbekistan; 2 Karshi State Technical University, Qarshi, Uzbekistan; 3 Bukhara State Medical Institute, Bukhara, Uzbekistan

**Keywords:** embeddedness, gender, informality, mahalla, market infrastructure, small entrepreneurship, social inclusion, social infrastructure

## 1 Introduction

Uzbekistan has moved further and faster on small-business reform than most countries in the post-Soviet region. Between 2017 and 2025 the share of small business in GDP rose from roughly one third to over half, and the number of registered small entities reached 1.21 million by October 2025 (State Statistics Agency of the Republic of Uzbekistan, 2025). The country was recognized as one of the five largest global improvers in the 2024 Women, Business and the Law index, with an 11.9-point gain taking its score to 82.5 (World Bank, 2024). The Uzbekistan-2030 strategy targets a doubling of exports and an increase in the number of exporting enterprises from roughly 5,700 to 15,000 (Ministry of Investment and Foreign Trade of the Republic of Uzbekistan, 2024).

These figures have been read almost exclusively through an economic and engineering lens. The dominant policy frame treats market infrastructure as a matter of roads, customs procedures, payment systems, credit lines, and digital platforms. That frame is not wrong. It is incomplete in a specific and consequential way, and the way it is incomplete is sociological.

This article makes a conceptual argument rather than reporting new data. Our contribution is not the observation that markets are socially embedded, which has been established since Polanyi (1944) and Granovetter (1985). It is threefold and more specific.

First, we argue that the social layer of market infrastructure should be treated as infrastructure in its own right, not as context surrounding the “real” infrastructure. This extends to a post-Soviet setting the argument developed by Elyachar (2010) for Cairo, where the communicative channels produced by women's everyday sociality function as economic infrastructure in the same register as roads and telephone lines, and it connects that argument to Star's (1999) and Larkin's (2013) insistence that infrastructure is relational and becomes visible only through the practices that sustain or fail to sustain it.

Second, we specify the mechanism by which social structure acts on formally universal infrastructure. We call it differential access filtering, and we decompose it using the four sources of social capital distinguished by Portes and Sensenbrenner (1993), namely value introjection, bounded solidarity, reciprocity exchanges, and enforceable trust. Two of these enable market participation and two constrain it, which gives the framework an internal reason to expect both positive and negative effects rather than asserting one or the other.

Third, we propose Uzbekistan as a scope condition for embeddedness theory. Most of the informality and embeddedness literature has examined settings where formal institutions were weak, absent or decaying (Mair and Martí, 2009; Williams and Shahid, 2016). Uzbekistan presents the inverse configuration, in which formal market infrastructure is being built rapidly and from above onto a dense, legally codified communal institution. The theoretical question this raises is whether embeddedness under rapid state-led formalization produces bridging or closure, and our answer is that it systematically produces both, with the balance determined by which of the four mechanisms a given reform activates.

The article proceeds as follows. Section 2 develops the theoretical framing. Section 3 sets out the five-layer framework and its social mediators. Section 4 reads the mahalla symmetrically. Section 5 applies the framework differentially to five entrepreneurial positions. Section 6 states explicitly what the available evidence does and does not support. Section 7 discusses feasibility of the policy implications. Section 8 sets out the theoretical contribution, limitations, testable propositions, and research agenda.

## 2 Market infrastructure as a sociological object

Infrastructure studies in sociology and anthropology have moved decisively away from treating infrastructure as a stock of physical assets. Star (1999) argued that infrastructure is relational rather than substantive, that it is embedded in other structures and practices, and that its distributional properties become visible mainly at the point of breakdown. Larkin (2013) extended this by treating infrastructures as objects that carry political and aesthetic meaning as well as goods, and by showing that the promise attached to an infrastructure is part of what it does. Graham and Marvin (2001) documented the process by which networked infrastructure, far from producing uniform access, splinters into premium and residual circuits.

Elyachar (2010) took the argument one step further in a way that is directly relevant here. Studying microfinance and empowerment projects in Cairo, she showed that the communicative channels generated by women's routine sociality constitute a social infrastructure that carries economic traffic as surely as a road does, and that development interventions make this infrastructure visible precisely by attempting to capture or format it. We take this as our starting point. If social ties are infrastructure, then a five-layer account of market infrastructure is not a four-layer account plus context. It is an account of five infrastructures, one of which happens to be made of relationships.

Economic sociology supplies the mechanism. Granovetter (1985) established that economic action is embedded in concrete networks of relations. Portes and Sensenbrenner (1993) then did the analytical work that the embeddedness literature most often omits, distinguishing four mechanisms by which social structure shapes economic action and, crucially, identifying their positive and negative consequences together. Value introjection and bounded solidarity generate commitment and mutual support but also generate demands for conformity. Reciprocity exchanges and enforceable trust lower transaction costs but also impose obligations that can drain the successful and restrict the individual autonomy on which enterprise growth depends. Portes and Landolt (1996) and Portes (1998) developed the resulting downsides, namely exclusion of outsiders, excess claims on group members, restrictions on individual freedom and downward leveling norms. We adopt this four-mechanism decomposition as the analytical core of our framework, because it is what allows a symmetrical reading of communal institutions rather than a celebratory one.

Two further strands complete the framing. The contextualization programme in entrepreneurship research insists that where and when entrepreneurship happens is constitutive rather than incidental (Welter, 2011; Welter et al., 2019; Kloosterman, 2010) mixed embeddedness perspective shows how the resources an entrepreneur can mobilize from social networks interact with the opportunity structure that markets and regulation supply. Meanwhile, the entrepreneurial ecosystem literature has become the dominant policy vocabulary for the phenomena we describe, and its own systematic reviews concede that it remains largely atheoretical and built on advanced-economy assumptions, with resource scarcity, structural gaps and institutional voids distinguishing emerging-economy ecosystems (Cao and Shi, 2021; Wurth et al., 2022). Our framework is intended in part as a sociological supplement to that literature, replacing its metaphor of an ecosystem of actors with an account of layered infrastructures and the filters that regulate access to them.

## 3 Five layers and five filters

By market infrastructure we mean the bundle of arrangements that allow a small firm to find customers, set prices, secure finance, manage logistics, and enforce rights. We distinguish five layers. The physical layer covers roads, warehouses, bazaars, and border posts. The financial layer covers banks, microfinance, payment instruments, and credit lines. The digital layer covers e-commerce, platform identifiers, e-government, and data systems (Nambisan, 2017; Bonina et al., 2021). The institutional layer covers registration, taxation, standards, and dispute resolution. The social layer covers trust networks, reputational systems, professional associations, rotating savings groups, and the mahalla.

The analytically important claim is not the existence of five layers but the relation between them. Access to any of the first four layers is mediated through the fifth, and the fifth distributes that access unequally. A credit assessment reads a reputation that is produced in a network. A customs procedure is navigated with the help of brokers and peers who have navigated it before. A digital marketplace is adopted first by entrepreneurs whose existing ties already include early users, which is why platform adoption tends to follow pre-existing network structure rather than to disrupt it (Bonina et al., 2021).

Where public investment addresses the first four layers without engaging the fifth, returns are eroded by filtering rather than by underprovision. The new road serves the bazaar of the better connected. The new credit line reaches firms with formal collateral. The new platform recruits younger urban men with smartphones and foreign-language skills. This is the splintering dynamic that Graham and Marvin (2001) described, produced here by social rather than commercial sorting.

Urban evidence from Uzbekistan supports this reading at the level of the layers themselves. Farmanov et al. (2025), examining smart-city implementation in Bukhara, find that deployment of sensors, platforms, and digital governance tools has run ahead of the inclusivity and citizen-engagement components, so that a technically advancing infrastructure is not correspondingly more usable. Dushanova et al. (2025) document the same asymmetry in the city's tourism infrastructure, where heritage sites, transport, and wayfinding remain inaccessible to people with mobility impairments despite a national policy commitment to barrier-free provision. Both cases show a filter that is not social in the network sense but produces the same result, namely infrastructure that is formally universal and practically selective. They also support treating market infrastructure as a sociotechnical assembly rather than a technical one, which is the premise of Section 2.

In the Uzbek case we identify five social mediators through which the filtering runs, namely mahalla networks, gender norms and the unpaid care burden, the urban-rural divide, the informal economy, and labor migration (Figure 1). These are not causes in a statistical sense and we do not claim to have estimated their effects. They are the channels through which formally universal infrastructure becomes differentially accessible, and Section 6 states precisely how much evidence stands behind each.

**Figure 1:**
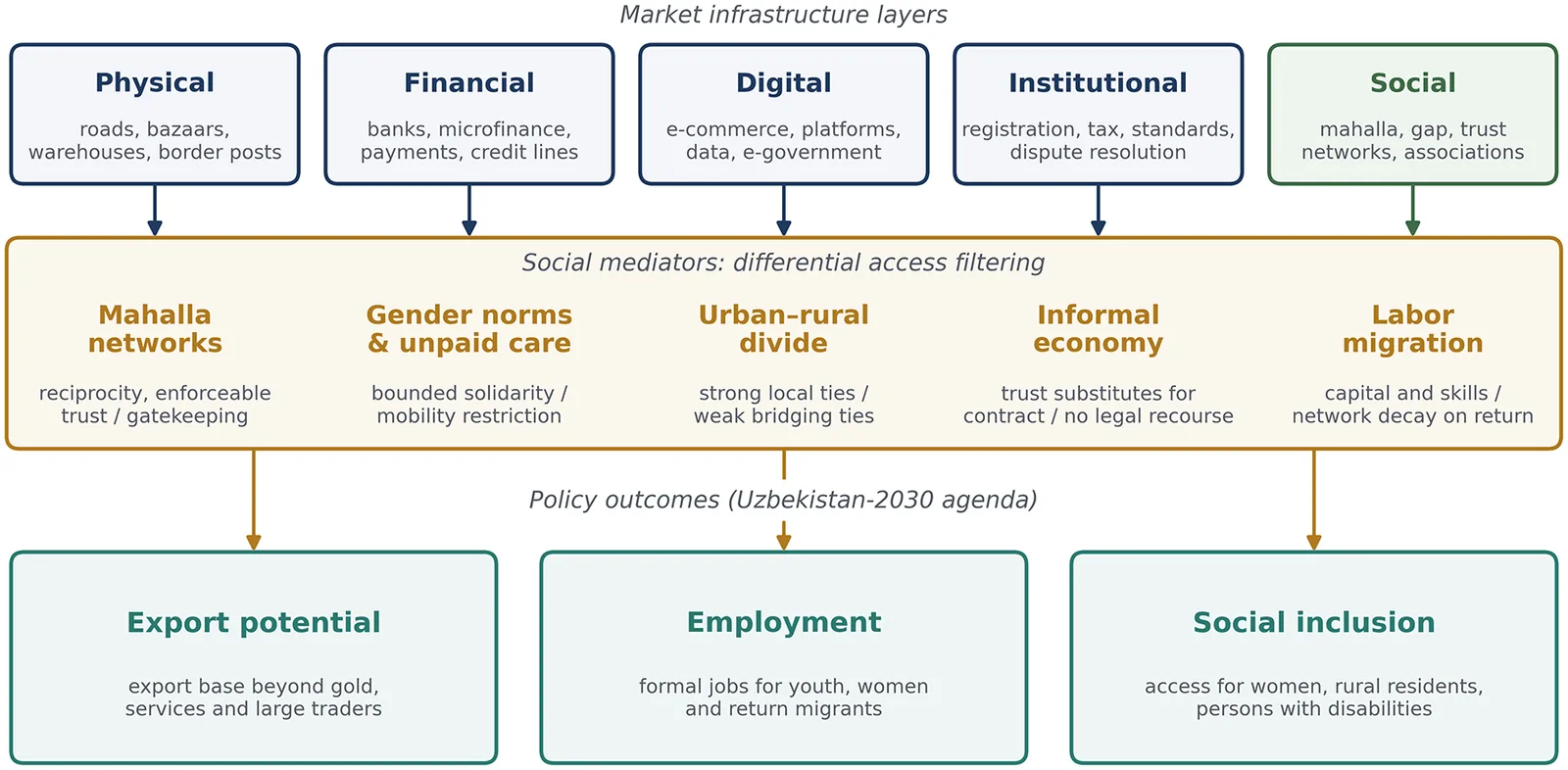
Five-layer framework of market infrastructure for small entrepreneurship in Uzbekistan. Infrastructure layers reach policy outcomes only through social mediators. Each mediator has an enabling and a restricting side, corresponding to the four mechanisms of social capital in (Portes and Sensenbrenner 1993). Which side dominates depends on the mechanism a given reform activates.

## 4 The mahalla read symmetrically

The mahalla is the strongest case and the hardest one. There are roughly nine to twelve thousand mahallas in Uzbekistan, each covering a few hundred to a few thousand residents, with a legal status as a unit of citizens' self-governance and an established role in welfare targeting and policy implementation (Urinboyev and Eraliev, 2022; Noori, 2006). Our earlier draft emphasized its enabling functions. The editorial assessment that this was one-sided is correct, and the theoretical framework we adopt in Section 2 requires a symmetrical reading, because Portes and Sensenbrenner's four mechanisms generate enabling and restricting effects from the same structure.

On the enabling side, the scholarly record documents the mahalla as a site of reciprocity exchanges and enforceable trust. Informal savings and rotating credit practices, locally called *gap*, circulate capital where formal credit does not reach, and community-based networks mediate economic activity through trust and reciprocity rather than legal contract (UNDP, 2025). Dadabaev (2013) shows that mahalla solidarity has historically rested on residence rather than kinship or ethnicity, which gave it an unusually broad reach across social groups. Urinboyev and Eraliev (2022) describe the mahalla as a legally plural arena in which competing orders coexist and produce new organizational forms, some of them generative.

On the restricting side, the record is at least as substantial and considerably older than our earlier draft acknowledged. Three findings matter for market infrastructure specifically.

First, the mahalla's incorporation into public administration has converted an association of residents into an instrument of state administration and, in the assessment of a large body of work, of social control (Urinboyev, 2011; Noori, 2006; Human Rights Watch, 2003). Urinboyev and Eraliev (2022) situate this within broader debates on how non-democratic states neutralize civil-society institutions through administrative and legal incorporation. An entrepreneur's relationship with the mahalla is therefore not a purely horizontal relationship among peers. It carries a vertical dimension, and any policy that routes market infrastructure through the mahalla routes it through that vertical dimension too.

Second, mahalla-based discretionary allocation has documented exclusionary effects along gender lines. Kamp (2004) shows that the delegation of welfare targeting to mahalla committees, staffed by untrained neighbors exercising discretion, placed divorced and abandoned women at particular risk of exclusion, and that solidarity among women in a mahalla cannot be assumed. This is bounded solidarity and enforceable trust operating exactly as Portes and Sensenbrenner predicted, producing support for insiders and exclusion at the boundary. If discretionary neighborhood assessment produces this pattern in welfare, there is no principled reason to expect a different pattern when the same bodies certify cooperatives, endorse borrowers or vouch for exporters.

Third, the conformity costs fall unevenly. Restrictions on individual autonomy and downward leveling norms are the two downsides of social capital most directly relevant to enterprise growth (Portes, 1998; Portes and Landolt, 1996). Ethnographic work on women traders in Uzbek bazaars describes exactly this tension, with women negotiating and renegotiating patriarchal norms rather than simply being enabled or simply being blocked by them (Kandiyoti, 2007).

The implication for policy is that “recognize the mahalla” is an underspecified instruction. What matters is which mechanism a given reform activates. Instruments that work through reciprocity exchange, such as linking *gap* groups to formal microfinance, plausibly extend access. Instruments that work through discretionary gatekeeping, such as making mahalla endorsement a condition of registration, credit or certification, plausibly reproduce existing exclusions and add a vertical dependency. We return to this distinction in Section 7.

## 5 Differentiated application: five entrepreneurial positions

A framework that applies identically to everyone explains nothing about distribution. Table 1 applies the five layers and their filters to five entrepreneurial positions whose constraints differ, which also responds to the reasonable objection that “small entrepreneurship” is not a single category.

**Table 1:** Differentiated application of the framework to five entrepreneurial positions.

Position	Binding layer	Dominant filter	Mechanism	Helps	Backfires
Women entrepreneurs	Financial, social	Unpaid care burden; gendered mobility and contracting norms	Value introjection; bounded solidarity	Care services that relax the time constraint; credit not requiring male-held collateral	Discretionary neighborhood endorsement, which reproduces the exclusions in Kamp (2004)
Rural firms	Physical, digital	Distance from both bazaar and buyer networks	Bounded solidarity: strong local, weak bridging ties	Bridging instruments such as producer associations with external market interfaces	Platform-first strategies assuming connectivity and literacy
Informal operators	Institutional	Asymmetry between formal rules and local norms of legitimacy	Enforceable trust substituting for legal contract	Graduated, low-cost formalization with early visible benefit	Enforcement-first formalization
Return migrants	Financial, social	Network decay during absence; unrecognized foreign earnings	Reciprocity exchanges, partially lapsed	Recognition of remittance and savings histories in credit scoring	Generic start-up programmes ignoring re-entry
Small exporters	Institutional, social	Social distance from international buyer networks; cross-border trust deficit	Enforceable trust that does not travel	Export consortia and third-party certification substituting for personal guarantee	Corridor and logistics investment alone

Three points follow from the table. The binding constraint differs by position, so a single infrastructure package cannot be expected to have uniform effects. The same mechanism that helps one position hinders another, which is why a framework built on Portes and Sensenbrenner's symmetric account is more useful here than one built on social capital as an unambiguous good. And the reforms most likely to backfire are, in several rows, the reforms currently most favored, namely discretionary community endorsement and platform-first digitalisation.

Two of the three policy outcomes named in the title deserve explicit grounding, since the framework is meant to bear on them.

Export concentration is documented. In 2024 gold, services, and industrial goods together accounted for roughly 70 percent of total exports (Observatory of Economic Complexity, 2025; U.S. Department of Commerce, 2025), and the number of exporting enterprises stood at around 5,700 in mid-2024 against the 2030 target of 15,000 (Ministry of Investment and Foreign Trade of the Republic of Uzbekistan, 2024). The shortfall is usually read as a logistics and certification problem. Our framework suggests it is also a trust problem, since enforceable trust built on personal relationship and cash does not travel across borders and infrastructure that supplies no substitute concentrates gains among firms already connected to international buyers. The concentration is a fact; its attribution to social filtering is our proposition.

Employment is where the filters compound. Women's labor-force participation is approximately half that of men, and women perform more than twice the male average of unpaid care work per day (UNDP, 2025; Popova and Otrachshenko, 2026), which constrains the time and mobility available for enterprise before any infrastructure question arises. A substantial share of small-business activity, particularly in bazaars and rural household plots, sits outside formal labor statistics, so employment effects of infrastructure reform are partly unobservable in the data used to evaluate them. Smagulova and Goncalves (2023) find for Central Asia that women's motivations for business entry do not map cleanly onto the necessity and opportunity dichotomy imported from advanced economies, which is a further reason to expect uniform instruments to perform unevenly across the positions in Table 1.

The migration row deserves a note because it is the one with the clearest empirical anchor. Kakhkharov (2019), using household data from Uzbekistan, finds that remittance-receiving households invest in family business only when remittances are supplemented by sufficient other income or savings, which locates the binding constraint in financial depth rather than in remittance volume. Remittances reached USD 14.8 billion in 2024, with the large majority originating in Russia (U.S. Department of Commerce, 2025). The policy inference commonly drawn, that large remittance inflows will convert into small-business formation, does not follow from the evidence, and the finding suggests that instruments addressing collateral and credit history are more likely to work than instruments addressing transfer cost alone.

## 6 What the evidence supports

The editorial assessment that our earlier draft presented several relationships as established facts when they are not is well founded. Table 2 states the status of each principal claim. We adopt three categories, namely documented, where the claim rests on data or on converging scholarly findings; contested, where scholarship supports the claim but disputes its interpretation or magnitude; and propositional, where the claim is an argument advanced by this article and awaiting test.

**Table 2:** Status of the article's principal claims.

Claim	Status	Basis
Small business accounts for over half of GDP and 1.21 million registered entities	Documented	State Statistics Agency of the Republic of Uzbekistan, 2025
Uzbekistan improved substantially on Women, Business and the Law 2024	Documented	World Bank, 2024
Women's labor-force participation is far below men's, and unpaid care time far above	Documented	UNDP, 2025; Popova and Otrachshenko, 2026
Export structure is concentrated and the exporter count falls well short of the 2030 target	Documented	Ministry of Investment and Foreign Trade of the Republic of Uzbekistan, 2024; Observatory of Economic Complexity, 2025
The mahalla functions simultaneously as a support institution and as an instrument of administration and social control	Documented, with continuing debate on the balance	Kamp, 2004; Noori, 2006; Urinboyev, 2011; Urinboyev and Eraliev, 2022
Remittance inflows do not by themselves generate small-business formation	Documented for Uzbekistan	Kakhkharov, 2019
Informality persists where formal rules and locally held norms are misaligned	Documented across settings, not tested for Uzbekistan	Williams and Shahid, 2016; Salvi et al., 2023
Digital and physical infrastructure in Uzbek cities is deployed ahead of its accessibility and inclusivity provisions	Documented for Bukhara	Farmanov et al., 2025; Dushanova et al., 2025
Infrastructure investment that ignores social filters produces concentrated rather than broad-based gains	Contested	Consistent with Graham and Marvin (2001) and with the export-concentration pattern, but not established causally for Uzbekistan
Legal recognition of informal trust practices would increase small-firm access to formal infrastructure	Propositional	No Uzbek evaluation exists; formalization literature reports mixed results
Mahalla-anchored guarantee funds and cooperative ownership would distribute access more equitably	Propositional	Scholz (2016) on platform cooperativism; no evaluation in this setting

We draw two conclusions from Table 2. The descriptive base for the argument is reasonably firm. The causal and prescriptive claims are not, and should be read as propositions rather than findings. Section 8 restates the most important of them in testable form.

## 7 Policy implications and their feasibility

Three implications follow, and we state feasibility conditions rather than recommendations alone.

Ex-ante social-impact assessment of infrastructure investment. Large infrastructure projects in Uzbekistan already undergo environmental and economic appraisal, so the institutional machinery for ex-ante assessment exists. Adding a distributional module is an administrative extension rather than a new apparatus. Its feasibility depends on data that partly exists, namely regional statistics disaggregated by sex and by urban and rural residence, and partly does not, namely firm-level access data. The honest statement is that the instrument is administratively feasible and evidentially under-equipped, and that the second problem is tractable within a normal statistical work programme.

Selective engagement with the mahalla. Section 4 implies that the choice is not between recognizing and bypassing the mahalla but between mechanisms. Linking *gap* groups to formal microfinance works through reciprocity exchange and asks the mahalla to do what it demonstrably already does. Making mahalla endorsement a gate for credit, certification or registration works through discretionary gatekeeping and asks it to do what the record suggests it does unevenly. The first is feasible and low-risk. The second is administratively easy and carries the risk documented by Kamp (2004). Where endorsement is used, an appeal route outside the mahalla is the minimum safeguard, and whether such a route would function is itself an empirical question. Cooperative and mutual ownership of market infrastructure, including mahalla-anchored guarantee funds, is a third option in this space (Scholz, 2016), and it is untested in this setting.

Graduated formalization rather than enforcement. The institutional-asymmetry literature finds that informality persists where formal rules diverge from locally held norms of legitimacy, and that enforcement-led formalization does not close that gap (Williams and Shahid, 2016; Salvi et al., 2023). The review evidence describes formalization as a pathway with phases rather than a switch, which implies instruments that lower the cost of the first step and make the reciprocal benefit visible early. Feasibility here is high in design and low in political attractiveness, since the revenue return is slow.

We do not claim that these instruments would produce the three policy outcomes. We claim that they are consistent with the mechanism the framework identifies, and that they are specified sharply enough to be evaluated.

## 8 Discussion

### 8.1 Theoretical contribution

The article makes three contributions to sociological theory rather than to policy description.

It treats the social layer as infrastructure rather than as context, extending Elyachar's (2010) account of social infrastructure from an informal-economy setting to one of rapid state-led formalization, and connecting it to the layered-infrastructure vocabulary that policy actually uses. This matters because the two literatures currently do not meet. Infrastructure studies has the relational insight and little purchase on policy instruments. Infrastructure policy has the instruments and no account of relational access.

It specifies the filtering mechanism using Portes and Sensenbrenner's (1993) four sources of social capital, which yields a framework that predicts enabling and restricting effects from the same structure and therefore cannot be used to celebrate communal institutions by construction.

It identifies a scope condition. The embeddedness and informality literatures have largely examined weak or hollowed-out formal institutions (Mair and Martí, 2009; Williams and Shahid, 2016). Uzbekistan offers the inverse case of dense, legally codified communal institutions receiving rapid top-down formal infrastructure. Our claim is that this configuration produces bridging and closure simultaneously, and that the balance is determined by which of the four mechanisms a reform activates. That is a claim about the conditions under which embeddedness theory yields different predictions, and it is testable.

### 8.2 Testable propositions

P1. Adoption of new digital market infrastructure by small firms follows pre-existing network position more closely than it follows firm size, sector or measured digital literacy.

P2. Reforms that operate through reciprocity exchange, such as linking rotating savings groups to formal microfinance, widen access, whereas reforms that operate through discretionary community endorsement narrow it, with the strongest narrowing among women without stable household male co-signers.

P3. Where mahalla endorsement functions as a gate to formal infrastructure, exclusion concentrates among the same categories identified in welfare targeting, namely divorced, widowed and recently arrived households.

P4. Small-firm export entry responds more strongly to third-party certification and consortium membership, which substitute for personal guarantee, than to reductions in logistics cost of comparable value.

P5. Return-migrant business formation responds to recognition of remittance and savings histories in credit assessment rather than to remittance volume, consistent with Kakhkharov (2019).

P6. Graduated formalization with an early and visible reciprocal benefit produces higher sustained formalization than enforcement of equivalent administrative intensity.

### 8.3 Limitations

Three limitations bound the argument. First, this is a conceptual article and presents no original data. Its descriptive base comes from official statistics and international agency reports whose methodologies vary, and shadow-economy estimates in particular carry wide uncertainty. Second, the mahalla literature we rely on is uneven across periods, with the strongest critical work dating from the Karimov era and the reform period after 2016 less thoroughly studied, so the extent to which the documented control functions persist in current form is an open question that our argument flags rather than settles. Third, the five mediators we identify are not exhaustive and are not independent of one another, since gender norms, care burden and mahalla governance are mutually constituted rather than separable, and a framework that lists them as parallel filters simplifies that entanglement.

### 8.4 Research agenda

The propositions in Section 8.2 imply a research programme with three components. A network-informed survey of small firms that measures platform adoption alongside network position would test P1 and is feasible with existing survey infrastructure. A comparative study of mahallas with and without gatekeeping roles in credit or certification would test P2 and P3, and is the study we regard as most urgent because it bears directly on a live policy choice. Administrative-data evaluation of remittance-linked credit scoring, which is currently being piloted in Uzbekistan, would test P5 with an unusually clean design.

More broadly, Central Asia offers the sociology of markets a configuration it has rarely examined, namely dense communal institutions meeting fast, deliberate, and state-led market construction. Whether embeddedness under those conditions bridges or encloses is not a question that can be answered from the existing literature, most of which was developed in settings where the state was retreating rather than building. Uzbekistan is a good place to find out.

## References

[B1] Bonina C. Koskinen K. Eaton B. Gawer A. (2021). Digital platforms for development: foundations and research agenda. Inf. Syst. J. 31, 869–902. doi: 10.1111/isj.12326

[B2] Cao Z. Shi X. (2021). A systematic literature review of entrepreneurial ecosystems in advanced and emerging economies. Small. Bus. Econ. 57, 75–110. doi: 10.1007/s11187-020-00326-y

[B3] Dadabaev T. (2013). Community life, memory and a changing nature of mahalla identity in Uzbekistan. J. Eurasian Stud. 4, 181–196. doi: 10.1016/j.euras.2013.03.008

[B4] Dushanova Y. Kadirova S. Kurbanova M. Khaydarova D. (2025). Smart and inclusive tourism in Bukhara: towards accessibility in heritage city. Front. Sports Act. Living 7:1624663. doi: 10.3389/fspor.2025.1624663 41333798 PMC12665691

[B5] Elyachar J. (2010). Phatic labor, infrastructure, and the question of empowerment in Cairo. Am. Ethnol. 37, 452–464. doi: 10.1111/j.1548-1425.2010.01265.x

[B6] Farmanov E. Dushanova Y. Akhmatova M. Kurbanova M. Mutaliyeva L. Turobova H. (2025). Balancing heritage and innovation: a multi-dimensional framework for smart city implementation in historic urban centers, the case of Bukhara, Uzbekistan. E3S Web Conf. 648:03024. doi: 10.1051/e3sconf/202564803024

[B7] Graham S. Marvin S. (2001). Splintering Urbanism: Networked Infrastructures, Technological Mobilities and the Urban Condition. London: Routledge. doi: 10.4324/9780203452202

[B8] Granovetter M. (1985). Economic action and social structure: the problem of embeddedness. Am. J. Cult. Sociol. 91, 481–510. doi: 10.1086/228311

[B9] Human Rights Watch (2003). From House to House: Abuses by Mahalla Committees. New York, NY: Human Rights Watch.

[B10] Kakhkharov J. (2019). Migrant remittances as a source of financing for entrepreneurship. Int. Migr. 57, 37–55. doi: 10.1111/imig.12531

[B11] Kamp M. (2004). “Between women and the state: Mahalla committees and social welfare in Uzbekistan,” in The Transformation of Central Asia: States and Societies from Soviet Rule to Independence, ed. P. J. Luong (Ithaca, NY: Cornell University Press), 29–58. doi: 10.7591/9781501731334-004

[B12] Kandiyoti D. (2007). The politics of gender and the Soviet paradox: neither colonized, nor modern? Centr. Asian Surv. 26, 601–623. doi: 10.1080/02634930802018521

[B13] Kloosterman R. C. (2010). Matching opportunities with resources: a framework for analysing (migrant) entrepreneurship from a mixed embeddedness perspective. Entrep. Reg. Dev. 22, 25–45. doi: 10.1080/08985620903220488

[B14] Larkin B. (2013). The politics and poetics of infrastructure. Annu. Rev. Anthropol. 42, 327–343. doi: 10.1146/annurev-anthro-092412-155522

[B15] Mair J. Martí I. (2009). Entrepreneurship in and around institutional voids: a case study from Bangladesh. J. Bus. Ventur. 24, 419–435. doi: 10.1016/j.jbusvent.2008.04.006

[B16] Ministry of Investment and Foreign Trade of the Republic of Uzbekistan (2024). Uzbekistan-2030 Export Development Strategy. Tashkent: Government of Uzbekistan.

[B17] Nambisan S. (2017). Digital entrepreneurship: toward a digital technology perspective of entrepreneurship. Entrep. Theory Pract. 41, 1029–1055. doi: 10.1111/etap.12254

[B18] Noori N. (2006). Expanding state authority, cutting back local services: decentralization and its contradictions in Uzbekistan. Centr. Asian Surv. 25, 533–549. doi: 10.1080/02634930701210526

[B19] Observatory of Economic Complexity (2025). Uzbekistan Country Profile. Available online at: https://oec.world/en/profile/country/uzb (Accessed September 24, 2026).

[B20] Polanyi K. (1944). The Great Transformation: The Political and Economic Origins of Our Time. Boston, MA: Beacon Press.

[B21] Popova O. Otrachshenko V. (2026). Women's empowerment in Uzbekistan: challenges and opportunities for the labor market. (CAP Uzbekistan Working Paper No. 5). Central Asia Program, The George Washington University, Washington, DC, United States.

[B22] Portes A. (1998). Social capital: its origins and applications in modern sociology. Annu. Rev. Sociol. 24, 1–24. doi: 10.1146/annurev.soc.24.1.1

[B23] Portes A. Landolt P. (1996). The downside of social capital. Am. Prospect 26, 18–21.

[B24] Portes A. Sensenbrenner J. (1993). Embeddedness and immigration: notes on the social determinants of economic action. Am. J. Cult. Sociol. 98, 1320–1350. doi: 10.1086/230191

[B25] Salvi E. Belz F.-M. Bacq S. (2023). Informal entrepreneurship: an integrative review and future research agenda. Entrep. Theory Pract. 47, 265–303. doi: 10.1177/10422587221115365

[B26] Scholz T. (2016). Platform Cooperativism: Challenging the Corporate Sharing Economy. New York, NY: Rosa Luxemburg Stiftung.

[B27] Smagulova G. Goncalves M. (2023). Drivers for women entrepreneurship in Central Asia: a case analysis of Kazakhstani enterprises. J. Transnatl. Manag. 28, 1–55. doi: 10.1080/15475778.2023.2285621

[B28] Star S. L. (1999). The ethnography of infrastructure. Am. Behav. Sci. 43, 377–391. doi: 10.1177/00027649921955326

[B29] State Statistics Agency of the Republic of Uzbekistan (2025). Key Indicators of Small Entrepreneurship in the Republic of Uzbekistan, January–October 2025. Tashkent.

[B30] U.S. Department of Commerce (2025). Uzbekistan Country Commercial Guide. Washington, DC: International Trade Administration.

[B31] UNDP (2025). Women's Entrepreneurship in Uzbekistan: Assessment and Recommendations. Tashkent: United Nations Development Programme.

[B32] Urinboyev R. (2011). Law, social norms and welfare as means of public administration: case study of mahalla institutions in Uzbekistan. NISPAcee J. Public Adm. Policy 4, 33–57. doi: 10.2478/v10110-011-0002-8

[B33] Urinboyev R. Eraliev S. (2022). Informal civil society initiatives in non-Western societies: mahallas in Uzbekistan. Centr. Asian Surv. 41, 477–497. doi: 10.1080/02634937.2022.2058915

[B34] Welter F. (2011). Contextualizing entrepreneurship: conceptual challenges and ways forward. Entrep. Theory Pract. 35, 165–184. doi: 10.1111/j.1540-6520.2010.00427.x

[B35] Welter F. Baker T. Wirsching K. (2019). Three waves and counting: the rising tide of contextualization in entrepreneurship research. Small. Bus. Econ. 52, 319–330. doi: 10.1007/s11187-018-0094-5

[B36] Williams C. C. Shahid M. S. (2016). Informal entrepreneurship and institutional theory: explaining the varying degrees of (in)formalization of entrepreneurs in Pakistan. Entrep. Reg. Dev. 28, 1–25. doi: 10.1080/08985626.2014.963889

[B37] World Bank (2024). Women, Business and the Law 2024. Washington, DC: The World Bank. doi: 10.1596/978-1-4648-2063-2

[B38] Wurth B. Stam E. Spigel B. (2022). Toward an entrepreneurial ecosystem research program. Entrep. Theory Pract. 46, 729–778. doi: 10.1177/1042258721998948

